# Correlation between multi-parameter echocardiographic indicators and adverse pregnancy outcomes in women of advanced maternal age with pregnancy-induced hypertension syndrome: clinical application value

**DOI:** 10.3389/fmed.2026.1766747

**Published:** 2026-02-25

**Authors:** Jing Zhong, Kang Zhang

**Affiliations:** Department of Ultrasound, Xiangyang No. 1 People's Hospital, Hubei University of Medicine, Xiangyang, Hubei, China

**Keywords:** advanced maternal age, cardiac function, diastolic dysfunction, echocardiography, pregnancy outcome, pregnancy-induced hypertension

## Abstract

**Introduction:**

Hypertensive disorders of pregnancy are a major cause of maternal and perinatal morbidity and mortality, particularly among women of advanced maternal age.

**Materials and methods:**

This retrospective study evaluated the correlation between echocardiographic parameters and adverse pregnancy outcomes in 240 women aged ≥35 years diagnosed with pregnancy-induced hypertension syndrome (PIHS) from February 2019 to June 2022. Patients were classified into a favorable outcome group (*n* = 183) and an adverse outcome group (*n* = 57). Echocardiographic indicators—including ejection fraction (EF), stroke volume (SV), cardiac output (CO), E/A ratio, early diastolic mitral annular velocity (E′), E/E′ ratio, left atrial volume index (LAVI), pulmonary venous flow velocities (S-, D-, Ar-waves), and myocardial performance (Tei) index—were compared between groups.

**Results:**

Women with adverse outcomes had significantly lower EF, SV, CO, E/A ratio, and E′, while showing higher E/E′, LAVI, Ar-wave velocity, and Tei index, with reduced S- and D-wave velocities (all *p* < 0.001). A history of hypertension, nephritis, and a family history of hypertension were also identified as significant clinical risk factors. Multivariable logistic regression confirmed that both clinical and echocardiographic parameters were independently associated with poor outcomes. Receiver operating characteristic analysis demonstrated high discriminative power for key indices (AUC range: 0.78–0.90), and a combined model integrating multiple echocardiographic variables achieved an AUC of 0.925, indicating excellent predictive performance.

**Discussion:**

These findings suggest that multi-parameter echocardiographic assessment provides a reliable, non-invasive approach for identification of high-risk PIHS patients, supporting more effective monitoring and timely intervention to improve maternal and fetal prognosis.

## Introduction

Advanced maternal age (AMA) refers to pregnancy in women aged 35 years or older at the time of delivery. In recent decades, delayed childbearing has become increasingly common, leading to a notable rise in pregnancies among older women worldwide (1). Statistical data show that the birth rates for women aged 35–39 and 40–44 years have steadily increased across several regions, including China, Taiwan, and many high-income countries (2–9). For instance, the proportion of deliveries among women aged ≥35 years in Taiwan increased from 11.4% in 2003 to 19.1% in 2013 (6), and similar upward trends have been reported in Norway and the United Kingdom (8, 9). With advancing maternal age, the risk of pregnancy-related complications increases substantially (10, 11). Older pregnant women have higher rates of preterm birth, stillbirth, and postpartum hemorrhage compared with younger counterparts (12–14). They are also more likely to experience premature rupture of membranes and require cesarean delivery (14, 15). Among the complications of AMA, hypertensive disorders of pregnancy remain particularly important, representing one of the major non-obstetric causes of maternal and fetal morbidity and mortality. Hypertensive disorder complicating pregnancy (HDCP) accounts for an estimated 50,000–60,000 maternal deaths annually and remains a leading cause of perinatal mortality, particularly in low- and middle-income countries (16). Pregnancy-induced hypertension syndrome (PIHS) is a subtype of hypertensive disorders of pregnancy characterized by new-onset hypertension (systolic blood pressure ≥140 mmHg and/or diastolic blood pressure ≥90 mmHg) occurring after 20 weeks of gestation in previously normotensive women, with or without proteinuria, according to contemporary international and Chinese guidelines (2–4, 17, 18). Moreover, women who develop PIHS face a higher lifetime risk of cardiovascular disease.

Pregnancy involves extensive hemodynamic adaptations—including increased heart rate, elevated cardiac output, and reduced systemic vascular resistance—to accommodate maternal and fetal metabolic demands (1). In patients with PIHS, these adaptive mechanisms are disrupted, resulting in altered myocardial performance and ventricular remodeling. Accurate assessment of cardiac function is therefore essential for early diagnosis, risk stratification, and timely management. Echocardiography, a non-invasive and reproducible imaging modality, enables comprehensive evaluation of both systolic and diastolic cardiac function. Owing to its safety, accessibility, and diagnostic precision, echocardiography has become the preferred tool for assessing cardiovascular status in pregnancy (19). Recent studies have highlighted its value in identifying subclinical cardiac dysfunction in hypertensive disorders of pregnancy. However, few investigations have systematically explored the relationship between multi-parameter echocardiographic indices and adverse pregnancy outcomes in women of advanced maternal age with PIHS.

Therefore, the present study aimed to evaluate the correlation between various echocardiographic parameters and adverse pregnancy outcomes in women of advanced maternal age diagnosed with PIHS, and to explore the potential clinical application of echocardiographic assessment in assessing cardiac functional alterations associated with adverse pregnancy outcomes.

## Patients and methods

### Ethical approval

This retrospective study was approved by the Institutional Ethics Committee of Xiangyang No. 1 People's Hospital, Hubei University of Medicine, Hubei, China (Approval No. PHHUM/C25/H/19). Written informed consent for the use of anonymized clinical and echocardiographic data for research purposes was obtained at the time of hospitalization. The study was conducted in accordance with the ethical principles of the Declaration of Helsinki and relevant national guidelines for research involving human subjects.

### Study design and population

**Figure 1:**
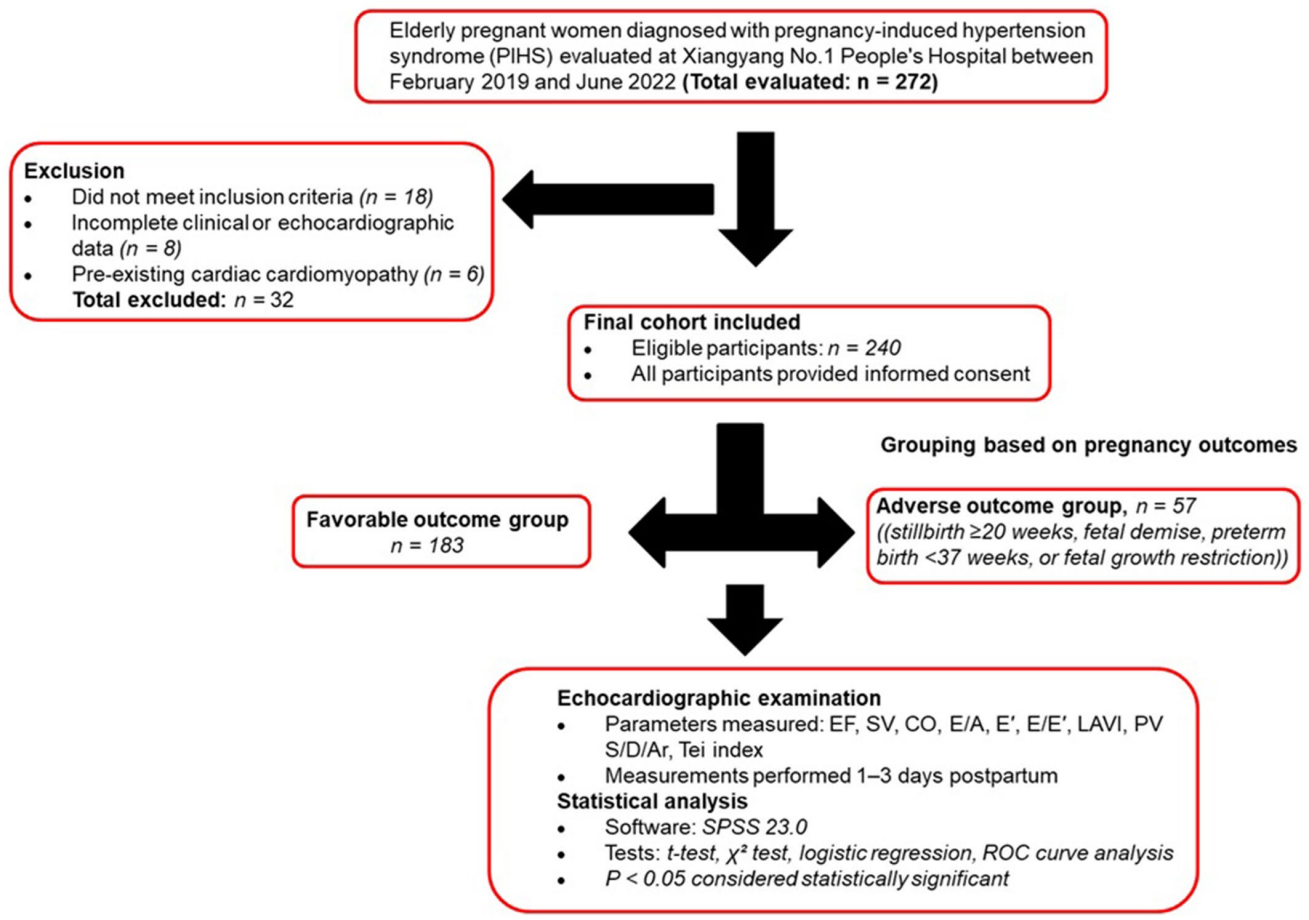
Study flow diagram. Flow diagram showing patient selection, grouping, and analysis in elderly women with pregnancy-induced hypertension syndrome (PIHS).

A total of 240 elderly pregnant women diagnosed with pregnancy-induced hypertension syndrome (PIHS) and treated at Xiangyang No. 1 People’s Hospital between February 2019 and June 2022 were retrospectively analyzed. Participants were divided according to pregnancy outcomes into a favorable outcome group (*n* = 183) and an adverse outcome group (*n* = 57). A schematic overview of the study design, screening, and analysis procedures is illustrated in Figure 1.

### Inclusion and exclusion criteria

Participants were enrolled based on strict eligibility conditions to ensure homogeneity of the study population and minimize confounding bias. Inclusion criteria were as follows: pregnant women aged over 35 years, with a singleton pregnancy, who had been clinically diagnosed with pregnancy-induced hypertension syndrome (PIHS) in accordance with international diagnostic criteria for hypertensive disorders of pregnancy (20). Only patients with complete clinical records and echocardiographic examination data available from the same hospitalization period were included. All diagnoses and echocardiographic assessments were confirmed by two senior obstetricians and a certified sonographer to maintain diagnostic accuracy and reproducibility.

Exclusion criteria were established to minimize confounding and ensure data reliability. Women were excluded if they had a history of psychiatric illness or cognitive impairment that could affect the accuracy of clinical data. Patients with severe uterine fibroids, ovarian cysts, or other gynecological conditions that could interfere with fetal growth or echocardiographic imaging were also excluded. Likewise, individuals with chronic infectious or systemic diseases, including HIV/AIDS, syphilis, chronic viral hepatitis, or autoimmune disorders, were excluded to reduce the influence of systemic inflammation on pregnancy outcomes. Patients with known structural heart disease, cardiomyopathy, congenital heart disease, significant valvular disease, or other pre-existing cardiac conditions affecting cardiac structure or function were excluded. Additional exclusion criteria included multiple gestations and gestational diabetes mellitus.

### Echocardiographic examination

All postpartum women underwent echocardiographic examination within 1–3 days after delivery or pregnancy termination. A Siemens ACUSON Oxana 2 color Doppler ultrasound system (probe frequency 2–4 MHz) was used. Examinations were performed in a quiet setting with the patient in the left lateral decubitus position by an experienced sonographer who was blinded to pregnancy outcomes. Each patient underwent a single standardized echocardiographic examination.

Standard echocardiographic parameters were acquired as follows:

*Structural indices:* Left atrial diameter (LAD), left ventricular end-diastolic diameter (LVDd), and interventricular septal end-diastolic thickness (IVSTd) measured from the parasternal long-axis view.

*Transmitral flow velocities:* Early diastolic (E wave) and atrial contraction (A wave) peak velocities recorded from the apical four-chamber view; the E/A ratio was calculated.

*Tissue Doppler imaging (TDI):* Early diastolic mitral annular velocity (E′) measured at the septal annulus, and the E/E′ ratio derived.

*Pulmonary venous flow:* Pulsed-wave Doppler sampling was performed with the sample volume positioned 1–2 cm proximal to the orifice of the upper pulmonary vein (right or left) in the apical four-chamber view to obtain systolic (S wave), diastolic (D wave), and atrial reversal (Ar wave) velocities. Measurements were acquired at end-expiration, and the mean value of three consecutive cardiac cycles was used for analysis.

*Myocardial performance index (Tei index):* Obtained from apical four-chamber view by measuring isovolumetric contraction time (IVCT), ejection time (ET), and isovolumetric relaxation time (IVRT), calculated as Tei index = (IVRT + IVCT) / ET.

*Cardiac output (CO):* Derived from stroke volume (SV) × heart rate (HR), where SV = velocity–time integral (VTI) of the left ventricular outflow tract × cross-sectional area.

*Ejection fraction (EF):* Calculated from end-diastolic (EDV) and end-systolic (ESV) ventricular volumes using EF = (EDV − ESV) / EDV × 100%.

Each measurement was repeated across three cardiac cycles, and mean values were used for analysis.

### Outcome assessment

Pregnancy outcomes were classified according to established obstetric definitions based on final delivery outcomes. Adverse pregnancy outcomes were predefined as events occurring at or beyond 20 weeks of gestation, including stillbirth (intrauterine fetal death ≥20 weeks), fetal demise, preterm birth (<37 weeks), and fetal growth restriction (estimated fetal weight <10th percentile for gestational age). Participants without any of these events were classified as having a favorable outcome. Echocardiographic assessments were performed postpartum, within 1–3 days after delivery or pregnancy termination, and the measured cardiac parameters were analyzed in relation to the documented pregnancy outcomes. Baseline demographic and clinical characteristics of the two groups are summarized in Table 1.

**Table 1:** Comparison of general and clinical characteristics between the favorable and adverse outcome groups.

Variable	Favorable outcome group (*n* = 183)	Adverse outcome group (*n* = 57)	*t*/*χ*^2^	*p*-value
Age (years)	36.22 ± 3.71	36.45 ± 3.68	0.401	0.689
BMI (kg/m^2^)	25.68 ± 2.17	25.82 ± 2.21	0.407	0.684
SBP (mmHg)	150.22 ± 11.31	151.38 ± 12.06	0.606	0.545
DBP (mmHg)	92.44 ± 9.05	93.08 ± 9.27	0.436	0.663
History of multiple pregnancies [*n* (%)]	32 (17.5)	12 (21.1)	0.391	0.532
Diabetes history [*n* (%)]	10 (5.5)	5 (8.8)	0.851	0.356
Hypertension history [*n* (%)]	12 (6.6)	11 (19.3)	8.142	0.004
Nephritis history [*n* (%)]	9 (4.9)	7 (12.3)	4.044	0.044
Family history of hypertension [*n* (%)]	24 (13.1)	15 (26.3)	6.048	0.014
Education years	12.35 ± 2.18	12.01 ± 2.15	0.985	0.326
Parity	1.54 ± 0.61	1.61 ± 0.64	0.711	0.478
Place of residence (urban/rural)	91/92	26/31	0.009	0.926

Data are presented as mean ± SD or number (percentage). *t*, independent-samples *t*-test; *χ*^2^, chi-square test.

### Clinical data collection

Demographic and obstetric data collected included maternal age, body mass index (BMI), systolic and diastolic blood pressures (SBP, DBP), gravidity and parity, years of education, and place of residence. Medical history variables included a history of pre-existing hypertension, diabetes mellitus, nephritis, and family history of hypertension (maternal or paternal). Information regarding antihypertensive treatment during pregnancy was extracted from medical records, including the use of standard obstetric antihypertensive medications administered according to institutional protocols. All clinical variables were obtained retrospectively from electronic medical records and obstetric charts and were recorded during the same hospitalization period as the echocardiographic examination.

### Statistical analysis

Data were analyzed using SPSS version 23.0 (IBM Corp., Armonk, NY, USA). Continuous variables were expressed as mean ± standard deviation (SD) and compared between groups using independent-samples *t*-tests. Categorical variables were presented as frequencies and percentages and analyzed using the *χ*^2^ test. Variables with *p* < 0.05 in univariate analyses were considered candidate predictors and entered into multivariable logistic regression models to identify independent risk factors for adverse pregnancy outcomes. Antihypertensive therapy during pregnancy was not included in the multivariable regression model because of its collinearity with blood pressure variables and to avoid further reduction in the events-per-variable ratio, which could increase the risk of model overfitting. To reduce the risk of overfitting, only variables with clear clinical relevance and statistical significance were retained in the final multivariable model. Baseline confounders, including history of hypertension, nephritis, and family history of hypertension, were incorporated as covariates. Model performance was evaluated using the Hosmer–Lemeshow goodness-of-fit test and Nagelkerke *R*^2^. Receiver operating characteristic (ROC) curve analysis was performed to assess the predictive accuracy of echocardiographic parameters. A two-sided *p*-value < 0.05 was considered statistically significant.

## Results

### Comparison of general patient data

Among the 240 elderly pregnant women with pregnancy-induced hypertension syndrome (PIHS), 183 experienced favorable pregnancy outcomes, whereas 57 had adverse outcomes. As summarized in Table 1, there were no significant differences between the two groups in terms of age, body mass index (BMI), systolic or diastolic blood pressure, history of multiple pregnancies, parity, years of education, or place of residence (all *p* > 0.05). However, patients in the adverse outcome group exhibited a significantly higher prevalence of pre-existing hypertension, nephritis, and a family history of hypertension (*p* < 0.05). These findings indicate that hereditary and chronic hypertensive conditions may predispose elderly pregnant women with PIHS to unfavorable pregnancy outcomes.

### Comparison of cardiac morphological indicators

Echocardiographic evaluation of structural cardiac indices revealed no statistically significant differences between the two groups in left ventricular end-diastolic diameter (LVDd), interventricular septal thickness (IVSTd), or left atrial diameter (LAD) (*p* > 0.05; Table 2). This suggests that the baseline chamber dimensions and wall thickness were not primary determinants of adverse outcomes in this cohort. The absence of structural differences indicates that functional rather than morphological cardiac alterations may contribute more prominently to PIHS-related complications.

**Table 2:** Comparison of cardiac morphological indicators between the favorable and adverse outcome groups (mean ± SD, mm).

Parameter	Favorable outcome group (*n* = 183)	Adverse outcome group (*n* = 57)	*t*	*p*-value
LVDd (mm)	46.27 ± 3.92	46.85 ± 3.87	1.023	0.307
IVSTd (mm)	9.12 ± 1.02	9.22 ± 0.98	0.610	0.542
LAD (mm)	33.81 ± 2.58	34.06 ± 2.63	0.568	0.571

No statistically significant differences were observed between groups (*p* > 0.05).

### Comparison of echocardiographic functional parameters

Functional echocardiographic indices differed significantly between the two groups (Table 3). The adverse outcome group demonstrated markedly lower ejection fraction (EF), stroke volume (SV), cardiac output (CO), E/A ratio, and early diastolic mitral annular velocity (E′) compared with the favorable outcome group (all *p* < 0.05). Conversely, E/E′ ratio and left atrial volume index (LAVI) were significantly higher in the adverse outcome group (*p* < 0.05). These alterations reflect diastolic dysfunction, impaired myocardial relaxation, and increased left atrial pressure load, which collectively suggest subclinical cardiac dysfunction as a contributing factor to poor maternal and fetal outcomes in elderly PIHS patients.

**Table 3:** Comparison of left ventricular functional parameters between the favorable and adverse outcome groups.

Parameter	Favorable outcome group (*n* = 183)	Adverse outcome group (*n* = 57)	*t*	*p*-value
EF (%)	65.83 ± 3.84	59.26 ± 4.02	11.518	<0.001
SV (mL)	73.15 ± 5.62	66.08 ± 6.01	8.083	<0.001
CO (L/min)	5.36 ± 0.63	4.78 ± 0.52	6.212	<0.001
E/A	1.16 ± 0.14	0.97 ± 0.13	9.389	<0.001
E′ (cm/s)	11.06 ± 1.18	9.74 ± 1.11	7.330	<0.001
E/E′	7.46 ± 0.71	8.59 ± 0.74	9.929	<0.001
LAVI (mL/m^2^)	25.88 ± 2.41	28.97 ± 2.35	8.443	<0.001

EF, ejection fraction; SV, stroke volume; CO, cardiac output; LAVI, left atrial volume index. All parameters showed significant differences between groups (*p* < 0.01).

### Comparison of pulmonary vein flow parameters and Tei index

As shown in Table 4, analysis of pulmonary venous flow dynamics revealed that the adverse outcome group had significantly lower pulmonary vein S-wave and D-wave velocities, but higher Ar-wave velocity and Tei index values compared with the favorable outcome group (all *p* < 0.05). These changes indicate impaired left atrial compliance and altered ventricular filling dynamics, implying that abnormalities in pulmonary venous flow patterns and an increased myocardial performance index are potential early markers of adverse pregnancy outcomes in PIHS.

**Table 4:** Comparison of pulmonary venous flow parameters and myocardial performance (Tei) index between groups.

Parameter	Favorable outcome group (*n* = 183)	Adverse outcome group (*n* = 57)	*t*	*p*-value
S-wave (cm/s)	49.15 ± 4.38	45.18 ± 4.06	6.110	<0.001
D-wave (cm/s)	47.26 ± 4.11	43.03 ± 3.98	6.447	<0.001
Ar-wave (cm/s)	29.12 ± 3.47	33.15 ± 3.65	7.014	<0.001
Tei index	0.46 ± 0.07	0.55 ± 0.08	8.623	<0.001

S, systolic wave; D, diastolic wave; Ar, atrial reversal wave; Tei, myocardial performance index.

### Multivariate logistic regression analysis of risk factors

To identify independent determinants of adverse pregnancy outcomes, variables with *p* < 0.05 in univariate analysis were entered into a multivariate binary logistic regression model (Table 5). A history of hypertension, nephritis, and a family history of hypertension emerged as significant independent clinical risk factors (*p* < 0.05). Among echocardiographic parameters, higher ejection fraction (EF), cardiac output (CO), E/A ratio, and early diastolic mitral annular velocity (E′) were independently associated with a *reduced* risk of adverse pregnancy outcomes (OR < 1), whereas higher E/E′ ratio and left atrial volume index (LAVI) were independently associated with an *increased* risk of adverse outcomes (OR > 1), indicating impaired systolic and diastolic cardiac function in patients with unfavorable outcomes. The direction of these associations is consistent with the between-group comparisons shown in Tables 3, 4. The final model included only the most significant clinical and echocardiographic variables to minimize overfitting. Antihypertensive therapy during pregnancy was not entered into the model because of collinearity with blood pressure variables and concerns regarding the events-per-variable ratio. It demonstrated good calibration (Hosmer–Lemeshow *p* = 0.63) and strong explanatory power (Nagelkerke *R*^2^ = 0.726), correctly classifying 88.3% of cases.

**Table 5:** Multivariate logistic regression analysis of factors associated with adverse pregnancy outcomes.

Variable	*B*	S.E.	Wald	*p*-value	OR	95% CI for OR
History of hypertension	1.214	0.385	9.955	0.002	3.366	1.624–6.978
History of nephritis	0.978	0.412	5.635	0.018	2.661	1.179–6.005
Family history of hypertension	0.864	0.378	5.218	0.022	2.372	1.132–4.969
Ejection fraction (EF, %)	−1.241	0.349	12.646	<0.001	0.289	0.147–0.568
Cardiac output (CO, L/min)	−1.874	0.488	14.760	<0.001	0.153	0.059–0.400
E/A ratio	−1.095	0.389	7.942	0.005	0.335	0.156–0.717
Early diastolic mitral annular velocity (E′, cm/s)	−1.213	0.426	8.092	0.004	0.297	0.130–0.680
E/E′ ratio	1.158	0.429	7.272	0.007	3.183	1.360–7.447
Left atrial volume index (LAVI, mL/m^2^)	1.256	0.466	7.251	0.007	3.512	1.408–8.762

Model fit: Nagelkerke *R*^2^ = 0.726; Hosmer–Lemeshow *χ*^2^ = 6.18, *p* = 0.63. The model correctly classified 88.3% of cases. Only clinically relevant variables that were statistically significant in univariate analyses were included in the multivariate model to reduce the risk of overfitting. Given the number of adverse events (*n* = 57), the events-per-variable ratio was relatively low; therefore, the results should be interpreted with appropriate caution.

### Receiver operating characteristic (ROC) curve analysis

**Figure 2:**
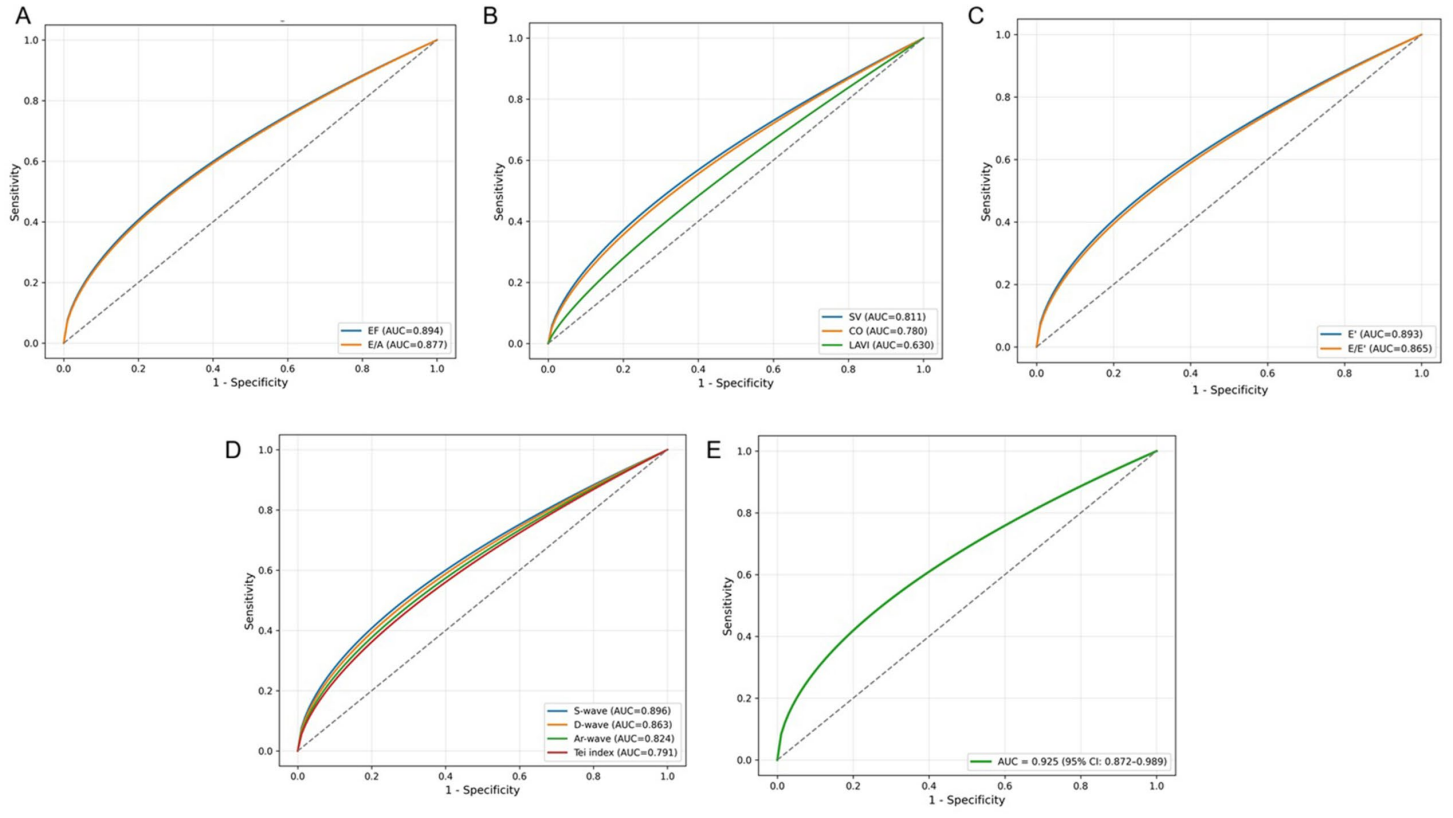
Receiver operating characteristic (ROC) curves for echocardiographic parameters predicting adverse pregnancy outcomes in women with pregnancy-induced hypertension syndrome (PIHS). **(A)** EF and E/A ratio; **(B)** SV, CO, and LAVI; **(C)** E′ and E/E′ ratio; **(D)** pulmonary vein S-, D-, Ar-waves, and Tei index; **(E)** combined diagnostic model integrating multiple echocardiographic parameters.

Receiver operating characteristic analysis was performed to evaluate the diagnostic utility of echocardiographic indices in predicting adverse pregnancy outcomes (Figures 2A–E). The area under the curve (AUC) values indicated strong predictive ability for several parameters: EF (AUC = 0.894, 95% CI 0.828–0.952), E/A ratio (AUC = 0.877, 95% CI 0.803–0.951), SV (AUC = 0.811, 95% CI 0.723–0.898), CO (AUC = 0.780, 95% CI 0.684–0.875), E′ (AUC = 0.893, 95% CI 0.826–0.959), and E/E′ ratio (AUC = 0.865, 95% CI 0.786–0.943). For pulmonary vein flow parameters, S-wave (AUC = 0.896, 95% CI 0.832–0.960), D-wave (AUC = 0.863, 95% CI 0.784–0.941), Ar-wave (AUC = 0.824, 95% CI 0.733–0.914), and Tei index (AUC = 0.791, 95% CI 0.695–0.888) also exhibited good discriminative value. Importantly, a combined diagnostic model integrating all echocardiographic parameters achieved an AUC of 0.925 (95% CI 0.872–0.989), reflecting excellent reflecting excellent discriminatory performance in distinguishing patients with adverse versus favorable pregnancy outcomes.

### Representative echocardiographic findings

**Figure 3:**
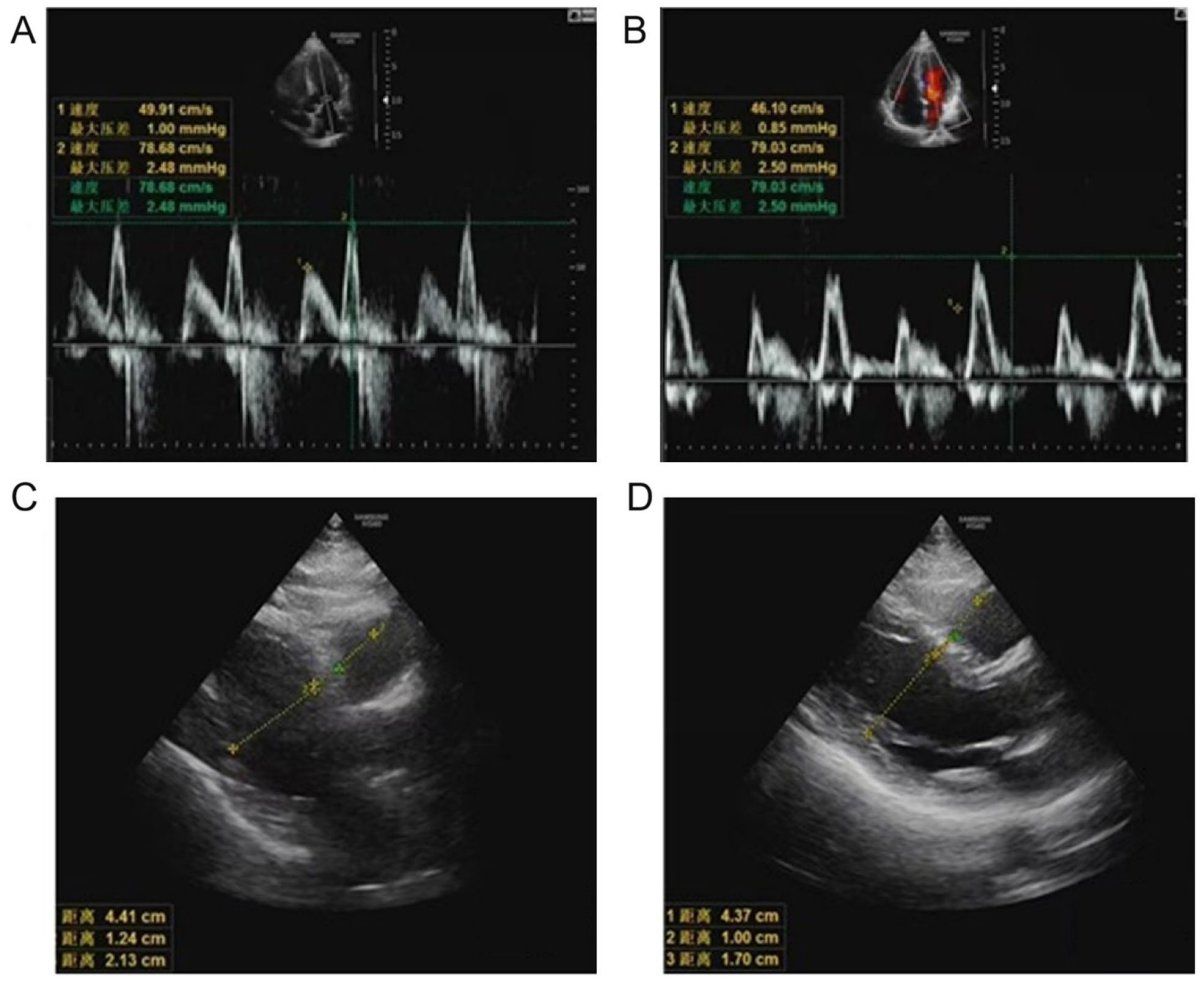
Doppler echocardiography images in women with pregnancy-induced hypertension syndrome (PIHS). **(A)** Mitral inflow velocity spectrum in a PIHS patient with an adverse pregnancy outcome. **(B)** Mitral inflow velocity spectrum in a PIHS patient with a favorable pregnancy outcome. **(C)** Left ventricular and left atrial diameters and wall thickness in a PIHS patient with an adverse outcome. **(D)** Left ventricular and left atrial diameters and wall thickness in a PIHS patient with a favorable outcome.

Representative echocardiographic images are shown in Figures 3A–E. Figure 3A shows a Doppler image of the mitral inflow velocity spectrum in a patient with an adverse pregnancy outcome, demonstrating attenuated E-wave and increased A-wave amplitude, findings suggestive of diastolic dysfunction. Figure 3B displays the corresponding image from a patient with a favorable pregnancy outcome, showing preserved diastolic filling and a normal E/A ratio. Figure 3C illustrates the echocardiographic assessment of left ventricular and atrial dimensions in a patient with an adverse outcome, revealing mild chamber dilation and increased left atrial volume. Figure 3D presents the same parameters in a patient with a favorable outcome, demonstrating normal chamber geometry and preserved systolic performance. Figure 3E provides a comparative composite image emphasizing the distinct functional patterns between favorable and adverse outcome groups. These findings collectively underscore the diagnostic relevance of functional echocardiographic indices—rather than purely structural measurements—in predicting pregnancy outcomes among elderly patients with pregnancy-induced hypertension syndrome (PIHS).

## Discussion

Pregnancy-induced hypertension syndrome (PIHS) is a pregnancy-specific disorder characterized by new-onset hypertension and proteinuria after 20 weeks of gestation in previously normotensive women. Affecting approximately 5–8% of pregnancies, it remains a major cause of maternal and fetal morbidity and mortality worldwide (21). The present study examined the association between echocardiographic parameters and adverse pregnancy outcomes in elderly women with PIHS, highlighting the clinical value of these indicators.

Our findings revealed no significant differences in structural echocardiographic parameters—left ventricular end-diastolic diameter (LVDd), interventricular septum end-diastolic thickness (IVSTd), and left atrial diameter (LAD)—between the favorable and adverse outcome groups. However, functional parameters showed marked disparities. The adverse outcome group demonstrated reduced ejection fraction (EF), stroke volume (SV), cardiac output (CO), E/A ratio, and early diastolic mitral annular velocity (E′), along with elevated E/E′ ratio and left atrial volume index (LAVI). Pulmonary venous flow analysis demonstrated significantly reduced S-wave and D-wave velocities, accompanied by increased Ar-wave velocity and elevated Tei index values in the adverse outcome group, indicating impaired left ventricular compliance and global myocardial dysfunction. PIHS is associated with widespread microvascular spasm, leading to increased peripheral resistance, elevated afterload, and progressive left ventricular hypertrophy, which ultimately impairs systolic and diastolic performance (22). These hemodynamic changes contribute to placental insufficiency, fetal growth restriction, preterm birth, and even intrauterine demise (23). Systemic vasoconstriction and endothelial dysfunction reduce placental perfusion, inducing intrauterine hypoxia and fetal cardiovascular stress. Prolonged cardiac overload further exacerbates myocardial injury and functional decline. Previous studies have reported similar findings, linking PIHS with abnormal cardiac remodeling and persistent cardiovascular risk (24–27). Rayes et al. (28) further emphasized that PIHS predisposes women to long-term cardiovascular disease, underscoring the importance of recognizing pregnancy-related cardiac functional alterations in women with PIHS.

Echocardiography provides a sensitive, non-invasive modality for evaluating these hemodynamic changes. Prior research indicates that PIHS patients exhibit increased systemic vascular resistance and reduced cardiac index due to elevated afterload (29, 30). Even after delivery, endothelial dysfunction and myocardial stress persist, predisposing patients to chronic cardiovascular morbidity (31, 32). Structural echocardiographic measurements alone are insufficient, as pregnancy itself induces physiological remodeling of preload and afterload (33, 34). Therefore, diastolic functional assessment is crucial. Diastolic dysfunction often precedes overt systolic failure and may exist despite preserved ejection fraction (35–37). Echocardiographic indicators such as E/A ratio, E′, E/E′ ratio, and LAVI reliably reflect ventricular relaxation and filling pressures (38–46). In this study, diastolic parameters (E/A ratio, E′, E/E′, and LAVI) and global performance indices (Tei index) demonstrated high predictive value for adverse outcomes. Elevated E/E′ and LAVI values indicate increased left atrial pressure and ventricular stiffness, while decreased E/A and E′ suggest impaired relaxation. These alterations reflect subclinical myocardial dysfunction and hemodynamic compromise that may contribute to placental hypoperfusion and fetal distress.

The pulmonary vein flow parameters further corroborated these findings. In the adverse outcome group, reduced S-wave and D-wave velocities together with elevated Ar-wave velocity suggested increased atrial pressure and impaired left ventricular compliance. The Tei index, a global measure of myocardial performance encompassing both systolic and diastolic function, was also higher in the adverse outcome group, reinforcing its clinical value in evaluating PIHS-related cardiac dysfunction (47, 48). Multivariate analysis confirmed that a history of hypertension, nephritis, and a family history of hypertension were significant independent clinical risk factors. Echocardiographic indicators reflecting both systolic and diastolic dysfunction, including reduced EF, CO, E/A ratio, and E′, together with elevated E/E′ and LAVI, were independently associated with adverse pregnancy outcomes. These parameters reflect impaired myocardial relaxation, increased filling pressure, and compromised ventricular performance, consistent with previous studies on cardiac dysfunction in pregnancy-induced hypertension. Receiver operating characteristic (ROC) curve analysis demonstrated high diagnostic performance, with area under the curve (AUC) values ranging from 0.780 to 0.925. The combined diagnostic model achieved an AUC of 0.925, underscoring the strong predictive utility of integrated echocardiographic assessment. These results are consistent with previous reports linking cardiac dysfunction to increased maternal and fetal morbidity in PIHS (49–55).

Despite its strengths, this study has several limitations. First, the sample size was relatively modest and derived from a single-center cohort, which may limit the generalizability of the findings. Regional, ethnic, and socioeconomic heterogeneity was not examined, and long-term maternal cardiovascular outcomes beyond the perinatal period were not assessed. Second, although efforts were made to limit model complexity, the number of adverse pregnancy events was relatively small in relation to the number of candidate predictors, resulting in a low events-per-variable ratio and a potential risk of model overfitting. Therefore, the reported associations should be interpreted with appropriate caution. In addition, although information on antihypertensive therapy during pregnancy was collected, medication use was not included in the multivariable analysis because of collinearity with blood pressure variables and concerns regarding the events-per-variable ratio; consequently, residual confounding related to treatment effects cannot be completely excluded. Finally, echocardiographic assessments were performed in the postpartum period, and future prospective studies incorporating serial antepartum and postpartum echocardiographic evaluations are warranted. Large, multicenter studies with more diverse populations and extended follow-up are needed to validate these findings and to further refine echocardiographic risk stratification strategies in women with pregnancy-induced hypertension syndrome (PIHS).

## Conclusion

Echocardiography serves as an effective, non-invasive tool for evaluating cardiac structure and function in elderly women with pregnancy-induced hypertension syndrome. Functional parameters, including EF, SV, CO, E/A ratio, E′, E/E′, LAVI, pulmonary vein flow indices, and Tei index, demonstrate significant predictive value for adverse pregnancy outcomes. Recognition of pregnancy-related cardiac functional alterations through echocardiography may contribute to improved postpartum cardiovascular assessment and longer-term risk evaluation in women with PIHS.

## Data Availability

The raw data supporting the conclusions of this article will be made available by the authors, without undue reservation.

## References

[1] Radparvar AA Vani K Fiori K Gupta S Chavez P Fisher M . Hypertensive disorders of pregnancy: innovative management strategies. JACC Adv. (2024) 3:100864. doi: 10.1016/j.jacadv.2024.100864, 38938826 PMC11198296

[2] Thomopoulos C Hitij JB De Backer T Gkaliagkousi E Kreutz R Lopez-Sublet M . Management of hypertensive disorders in pregnancy: a position statement of the European Society of Hypertension Working Group “hypertension in women”. J Hypertens. (2024) 42:1109–32. doi: 10.1097/HJH.0000000000003739, 38690949

[3] Scott G Gillon TE Pels A von Dadelszen P Magee LA. Guidelines—similarities and dissimilarities: a systematic review of international clinical practice guidelines for pregnancy hypertension. Am J Obstet Gynecol. (2022) 226:S1222–36. doi: 10.1016/j.ajog.2020.08.018 32828743

[4] Magee LA Smith GN Bloch C Côté AM Jain V Nerenberg K . Guideline no. 426: hypertensive disorders of pregnancy: diagnosis, prediction, prevention, and management. J Obstet Gynaecol Can. (2022) 44:547–71.e1. doi: 10.1016/j.jogc.2022.03.002 35577426

[5] Cordier AG Russo FM Deprest J Benachi A. Prenatal diagnosis, imaging, and prognosis in congenital diaphragmatic hernia. Semin Perinatol. (2020) 44:101563. doi: 10.1053/j.semperi.2019.07.002, 31439324

[6] Tsakiridis I Giouleka S Arvanitaki A Giannakoulas G Papazisis G Mamopoulos A . Gestational hypertension and preeclampsia: an overview of national and international guidelines. Obstet Gynecol Surv. (2021) 76:613–33. doi: 10.1097/OGX.0000000000000942, 34724074

[7] Tita AT Szychowski JM Boggess K Dugoff L Sibai B Lawrence K . Treatment for mild chronic hypertension during pregnancy. N Engl J Med. (2022) 386:1781–92. doi: 10.1056/NEJMoa2201295, 35363951 PMC9575330

[8] Gupta K Balyan K Lamba B Puri M Sengupta D Kumar M. Ultrasound placental image texture analysis using artificial intelligence to predict hypertension in pregnancy. J Matern Fetal Neonatal Med. (2022) 35:5587–94. doi: 10.1080/14767058.2021.1887847, 33596762

[9] La-Orpipat T Suwanrath C. Pregnancy outcomes of adolescent primigravida and risk of pregnancy-induced hypertension. J Obstet Gynaecol. (2019) 39:934–40. doi: 10.1080/01443615.2019.1581736, 31180254

[10] Metoki H Iwama N Hamada H Satoh M Murakami T Ishikuro M . Hypertensive disorders of pregnancy: definition, management, and out-of-office blood pressure measurement. Hypertens Res. (2022) 45:1298–309. doi: 10.1038/s41440-022-00965-6, 35726086 PMC9207424

[11] Benschop L Duvekot JJ Roeters van Lennep JE. Future risk of cardiovascular disease after hypertensive disorders of pregnancy. Heart. (2019) 105:1273–8. doi: 10.1136/heartjnl-2018-313453, 31175138 PMC6678044

[12] Hauspurg A Countouris ME Catov JM. Hypertensive disorders of pregnancy and future maternal health. Curr Hypertens Rep. (2019) 21:96 31776692 10.1007/s11906-019-0999-7 PMC7288250

[13] Agrawal A Wenger NK. Hypertension during pregnancy. Curr Hypertens Rep. (2020) 22:64. doi: 10.1007/s11906-020-01070-0, 32852628

[14] Lopes Perdigao J Lewey J Hirshberg A Koelper N Srinivas SK Elovitz MA . Furosemide for accelerated postpartum blood pressure recovery. Hypertension. (2021) 77:1517–24. 33550824 10.1161/HYPERTENSIONAHA.120.16133 PMC8099047

[15] Reddy S Jim B. Hypertension and pregnancy: management and future risks. Adv Chronic Kidney Dis. (2019) 26:137–45. doi: 10.1053/j.ackd.2019.03.017, 31023448

[16] American College of Obstetricians and Gynecologists. ACOG practice bulletin no. 203: chronic hypertension in pregnancy. Obstet Gynecol. (2019) 133:26–50. 10.1097/AOG.0000000000003020 30575676

[17] Battarbee AN Sinkey RG Harper LM Oparil S Tita ATN. Chronic hypertension in pregnancy. Am J Obstet Gynecol. (2020) 222:532–41. doi: 10.1016/j.ajog.2019.11.1243, 31715148

[18] Magee LA Khalil A von Dadelszen P. Pregnancy hypertension diagnosis and care in the COVID-19 era. Ultrasound Obstet Gynecol. (2020) 56:7–10. doi: 10.1002/uog.22115, 32506723 PMC7300934

[19] Dash SS Jena P Khuntia S Pathak M Rath SK. Uterine artery pulsatility index for predicting pregnancy-induced hypertension. Int J Gynaecol Obstet. (2021) 154:431–5. doi: 10.1002/ijgo.13545, 33326607

[20] Magee LA Brown MA Hall DR Gupte S Hennessy A Karumanchi SA . ISSHP classification and management recommendations. Pregnancy Hypertens. (2022) 27:148–69. 35066406 10.1016/j.preghy.2021.09.008

[21] Yu L Zhou Q Peng Q Zeng S Yang Z. Velocity vector imaging echocardiography and NT-proBNP study of fetal cardiac function in pregnancy-induced maternal hypertension. J Clin Ultrasound. (2019) 47:285–91. doi: 10.1002/jcu.22720 30883813

[22] Febres-Cordero DA Young BC. Hypertensive disorders of pregnancy. NeoReviews. (2021) 22:e760–6. doi: 10.1542/neo.22-11-e760 34725140

[23] Martin SR Edwards A. Pulmonary hypertension and pregnancy. Obstet Gynecol. (2019) 134:974–87. doi: 10.1097/AOG.0000000000003549, 31599832

[24] Gencheva D Nikolov F Uchikova E Mihaylov R Pencheva B Vasileva M. IL-6 and echocardiographic correlations in hypertensive pregnancy. Cardiovasc J Afr. (2022) 33:65–73. doi: 10.5830/CVJA-2021-040, 34546286 PMC9364472

[25] Briller JE. Echocardiographic screening in hypertensive pregnancy disorders. J Am Coll Cardiol. (2022) 80:1477–9. doi: 10.1016/j.jacc.2022.08.717, 36202537

[26] Bromfield SG Ma Q DeVries A Inglis T Gordon AS. Maternal and neonatal outcomes in hypertensive pregnancy. BMC Pregnancy Childbirth. (2023) 23:514. 37452285 10.1186/s12884-023-05818-9 PMC10347833

[27] Castleman JS Ganapathy R Taki F Lip GY Steeds RP Kotecha D. Echocardiographic structure and function in hypertensive disorders of pregnancy: a systematic review. Circ Cardiovasc Imaging. (2016) 9:e004888 doi: 10.1161/CIRCIMAGING.116.004888 27609819

[28] Rayes B Ardissino M Slob EAW Patel KHK Girling J Ng FS. Association of hypertensive disorders of pregnancy with future cardiovascular disease. JAMA Netw Open. (2023) 6:e230034. doi: 10.1001/jamanetworkopen.2023.0034 36800181 PMC9938428

[29] Timor-Tritsch IE D’Antonio F Calí G Palacios-Jaraquemada J Meyer J Monteagudo A. Early first-trimester transvaginal ultrasound after cesarean delivery. Ultrasound Obstet Gynecol. (2019) 54:156–63. doi: 10.1002/uog.20225, 30677186

[30] Ardissino M Slob EAW Rajasundaram S Reddy RK Woolf B Girling J . Safety of beta-blocker and calcium channel blocker antihypertensive drugs in pregnancy: a Mendelian randomization study. BMC Med. (2022) 20:288. doi: 10.1186/s12916-022-02483-1 36064525 PMC9446737

[31] Lin X Lu C Ma G. Tissue Doppler imaging and myocardial strain in pregnancy-induced hypertension. Sci Rep. (2023) 13:21315. doi: 10.1038/s41598-023-48599-z 38044364 PMC10694130

[32] Hanchard TJ de Vries BS Quinton AE Sinosich M Hyett JA. The value of TDI combined with myocardial strain parameters in quantitative evaluation of left heart function in parturient with pregnancy-induced hypertension. Ultrasound Obstet Gynecol. (2020) 55:629–36. doi: 10.1002/uog.21962, 31909523

[33] Lopez AG Dominiczak AF Touyz R Schlaich M de Freminville JB Amar L. Hypertension with negative MIBG scintigraphy. Hypertension. (2022) 79:474–8. doi: 10.1161/HYPERTENSIONAHA.121.18012, 34879700

[34] Sørensen A Sinding M. Preeclamptic placenta and MRI insights. Hypertension. (2020) 75:1412–3. doi: 10.1161/HYPERTENSIONAHA.120.14855, 32401645

[35] Antza C Stabouli S Kotsis V. Practical guide for hypertensive disorders during pregnancy. J Hypertens. (2022) 40:1257–64. doi: 10.1097/HJH.0000000000003194, 35762468

[36] Luo TT Dong HM. Echocardiographic techniques for fetal cardiac function. Chin J Med Imaging Technol. (2018) 34:1299–302.

[37] Tolcher MC Fox KA Sangi-Haghpeykar H Clark SL Belfort MA. Labetalol versus nifedipine for acute hypertension. Am J Obstet Gynecol. (2020) 223:441.e1–8. doi: 10.1016/j.ajog.2020.06.018 32544404

[38] Jones EJ Hernandez TL Edmonds JK Ferranti EP. Postpartum follow-up disparities after hypertensive pregnancy. J Perinat Neonatal Nurs. (2019) 33:136–48. doi: 10.1097/JPN.0000000000000399, 31021939 PMC6485948

[39] Montaguti E Di Donna G Youssef A Pilu G. Maternal hemodynamics in hypertensive pregnancy. J Med Ultrason. (2022) 49:405–13. doi: 10.1007/s10396-022-01225-3, 35705778

[40] Cui H Wu Y Li Y. Echocardiographic evaluation of inferior vena cava compression. Chin J Med Ultrasound. (2020) 17:880.

[41] Giorgione V Melchiorre K O’Driscoll J Khalil A Sharma R Thilaganathan B. Maternal echocardiographic changes in twin pregnancy. Ultrasound Obstet Gynecol. (2022) 59:619–26. doi: 10.1002/uog.24852, 35000243

[42] Chou JC. Hypertensive pregnancy and cardiovascular risk. J Am Coll Cardiol. (2022) 79:2412–4. doi: 10.1016/j.jacc.2022.04.013, 35710192

[43] Tairy D Weiner E Kovo M Zamir AM Gandelsman E Levy M . Fetal growth restriction in hypertensive vs. heavy smoking women-placental pathology, ultrasound findings, and pregnancy outcomes. Reprod Sci. (2021) 28:819–27. doi: 10.1007/s43032-020-00373-6 33140325

[44] Stefano GT Zhao H Schluchter M Hoit BD. Assessment of echocardiographic left atrial size: accuracy of M-mode and two-dimensional methods and prediction of diastolic dysfunction. Echocardiography. (2012) 29:379–84. doi: 10.1111/j.1540-8175.2011.01643.x 22380762

[45] Nagueh SF Appleton CP Gillebert TC Marino PN Oh JK Smiseth OA . Recommendations for the evaluation of left ventricular diastolic function by echocardiography. J Am Soc Echocardiogr. (2009) 22:107–33. doi: 10.1016/j.echo.2008.11.023 19187853

[46] Nagueh SF. Non-invasive assessment of left ventricular filling pressure. Eur J Heart Fail. (2018) 20:38–48. doi: 10.1002/ejhf.971, 28990316

[47] Tei C Ling LH Hodge DO Bailey KR Oh JK Rodeheffer RJ . New index of myocardial performance. J Cardiol. (1995) 26:357–66. 8558414

[48] Pewowaruk RJ Racine J Iruretagoyena JI Roldán-Alzate A. Vascular shear stress in preeclampsia. Cardiovasc Eng Technol. (2020) 11:760–8. doi: 10.1007/s13239-020-00488-6, 33025370 PMC9021976

[49] Bangi EF Yousuf MH Upadhyay S Jain P Jain R. Review of hypertensive disorders of pregnancy. South Med J. (2023) 116:482–9. doi: 10.14423/SMJ.0000000000001571, 37263611

[50] Kissas G Hwuang E Thompson EW Schwartz N Detre JA Witschey WR . Feasibility of vascular parameter estimation for assessing hypertensive pregnancy disorders. J Biomech Eng. (2022) 144:121011. doi: 10.1115/1.4055679 36128759 PMC9836050

[51] Pęksa M Kamieniecki A Gabrych A Lew-Tusk A Preis K Świątkowska-Freund M. Cadherin staining continuity in the trophoblastic basal membrane correlates with increased resistance in uterine arteries and proteinuria in patients with pregnancy-induced hypertension. J Clin Med. (2022) 11:668. doi: 10.3390/jcm11030668 35160120 PMC8836559

[52] Erkamp JS Geurtsen ML Duijts L Reiss IKM Mulders AGMGJ Steegers EAP . Associations of maternal early-pregnancy glucose concentrations with placental hemodynamics, blood pressure, and gestational hypertensive disorders. Am J Hypertens. (2020) 33:660–9. doi: 10.1093/ajh/hpaa070 32322887 PMC10868575

[53] Nobles CJ Mendola P Mumford SL Silver RM Kim K Andriessen VC . Preconception blood pressure and its change into early pregnancy: early risk factors for preeclampsia and gestational hypertension. Hypertension. (2020) 76:922–9. doi: 10.1161/HYPERTENSIONAHA.120.14875 32755413 PMC7456510

[54] Leavitt K Običan S Yankowitz J. Prevention of hypertensive disorders in pregnancy. Clin Perinatol. (2019) 46:173–85. doi: 10.1016/j.clp.2019.02.002, 31010554

[55] Nguyen TM Castro LC. Hypertensive disorders and depression in pregnancy: pregnancy complications and fetal versus neonatal outcomes. J Women's Health. (2019) 28:1451–3. doi: 10.1089/jwh.2019.8107 31730424

